# Surveillance of Ticks and Tickborne *Borrelia*, *Ehrlichia*, and *Rickettsia* spp., Texas, USA, 2014–2021

**DOI:** 10.3201/eid3208.251104

**Published:** 2026-08

**Authors:** Yan Zhang, Elizabeth A. Mitchell, Rebecca J. Kilgore, Michael S. Allen

**Affiliations:** University of North Texas Health Science Center, Fort Worth, Texas, USA

**Keywords:** Ticks, *Borrelia*, *Ehrlichia*, *Rickettsia*, vector-borne infections, tick-borne, bacteria, bacterial infection, zoonoses, Texas, United States

## Abstract

We report the temporal and geographic distribution of ticks and tickborne *Borrelia*, *Ehrlichia*, and *Rickettsia* spp. bacteria in Texas, USA, 2014–2021, updating the previous 2008–2014 passive surveillance report. We identified 9 tick species and an overall prevalence of tested bacterial species of 26.0%, predominantly *Rickettsia amblyommatis*.

Texas, USA, is home to several human-biting tick species that carry and transmit bacterial pathogens, including *Rickettsia*, *Borrelia*, and *Ehrlichia* spp. (*1*–*3*). Since 2004, the University of North Texas Health Tick-Borne Disease Laboratory (Fort Worth, TX, USA) has performed pathogen testing of ticks removed from humans and submitted to the Texas Department of State Health Services (*1*,*4*,*5*). Building on previous surveillance efforts, we further analyzed the temporal and geographic distributions of tick species and tickborne bacteria in Texas. The data reflected human–tick interactions rather than natural tick activities and distributions and could be useful in informing public health risk in Texas.

Entomologists at the Texas Department of State Health Services identified tick species morphologically. Unidentified specimens were classified by tick mitochondrial 16S rRNA gene analysis at University of North Texas Health. They used PCRs and Sanger sequencing (*1*) to determine the presence of bacteria: *Borrelia* by using the *flaB* gene target, *Ehrlichia* by using the *dsb* gene target, and *Rickettsia* by using the *rompA* gene target. We excluded 2020 data from the temporal analysis because of testing delays caused by the COVID-19 pandemic.

A total of 1,625 ticks (9 species) from Texas were tested from October 1, 2014, through September 30, 2021. The most frequently submitted tick species was *Amblyomma americanum* (48.9%), followed by *Dermacentor variabilis* (21.2%), *A*. *maculatum* (11.9%), *Ixodes scapularis* (10.0%), *Rhipicephalus sanguineus* (3.8%), and *A*. *mixtum* (3.4%) (Appendix Table 1). Nymphs represented a substantial proportion of *A. americanum* (42.8%) and *A. mixtum* submissions (60.0%), and larvae were 2.1% (*A. americanum*) and 3.6% (*A. mixtum*) of submissions. By contrast, the other submitted species were mostly adults. The female-to-male ratio was close to 1 (0.87–1.2) for most species, except for *I. scapularis* (90.7%) and *D. variabilis* (62.3%) tick submissions, which were mostly female.

Adult *A. americanum*, *A. maculatum*, *D. variabilis*, and *I. scapularis* tick submissions demonstrated clear temporal patterns (Figure 1, panel A; Appendix Table 2). Adult *A. americanum* submissions were much higher in May–July. We observed the highest adult *A. maculatum* tick submissions in August. Submission of adult *I. scapularis* ticks peaked in March, and *D. variabilis* ticks peaked in June. We observed no obvious temporal patterns in nymphal *A. americanum* or *A. mixtum* submissions (Figure 1, panel B).

**Figure 1:**
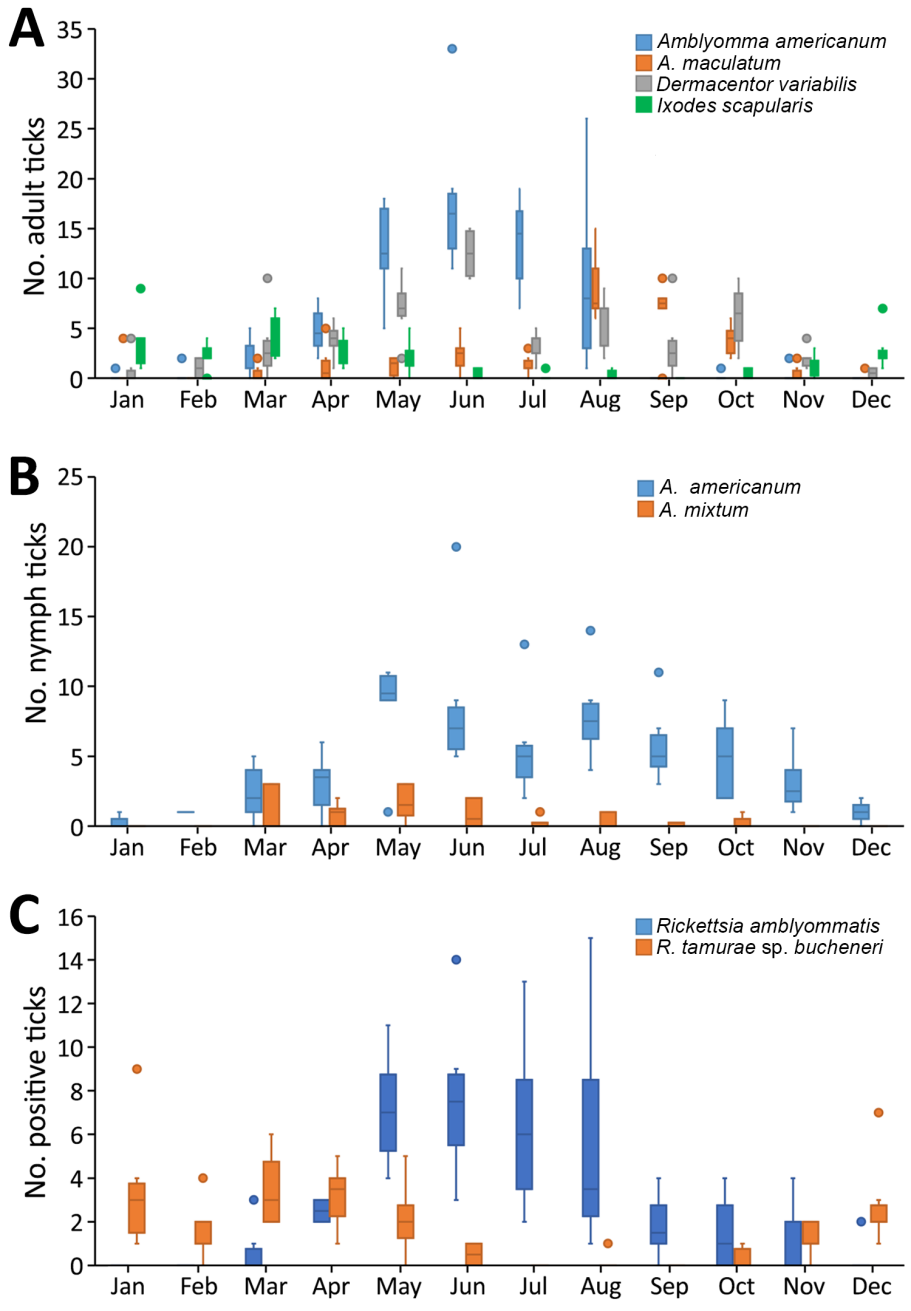
Box plot graph displaying temporal patterns of ticks submitted in Texas, USA, 2014–2021. A) Adult ticks; B) nymphs; C) *Rickettsia amblyommatis* and *R. tamurae* subspecies *buchneri*–positive ticks. Horizontal lines within boxes represent medians, box tops and bottoms indicate interquartile ranges (IQRs), and error bars represent minimum value within 1.5 × IQR below 25% and maximum value within 1.5 × IQR above 75%. Outlier points are data points <25% − 1.5 × IQR or >75% + 1.5 × IQR.

We identified 9 bacterial species from ticks and an overall prevalence rate of 26.0%, largely comprising spotted fever group rickettsiae. *Rickettsia amblyommatis* was the most common bacterium, with the following prevalence rates in each tick species: *A. mixtum*, 34.6%; *A. americanum*, 26.8%; *R. sanguineus*, 1.6%; *D. variabilis*, 0.58%; and *A. maculatum*, 0.5% (Appendix Table 1). *R*. *tamurae* subspecies *buchneri*, the second most frequent bacterial species, was detected only in *I. scapularis* ticks (85.2% positive rate, 90% in females, whereas all male ticks tested negative). *R. amblyommatis* and *R. tamurae* subsp. *buchneri*–positive ticks demonstrated temporal patterns similar to the overall submission patterns of *A. americanum* and *I. scapularis* ticks; peaks occurred from May–July for *R. amblyommatis* and in March for *R. tamurae* subsp. *buchneri* (Figures 1, panel C; Appendix Table 2). Lacking clinical evidence, the pathogenicity of *R. amblyommatis* is unclear. *R. tamurae* subsp. *buchneri* is thought to be a nonpathogenic endosymbiont of *I. scapularis* ticks, providing essential nutrients for tick survival (*6*). The presence of those 2 organisms might reduce the transmission of other pathogenic *Rickettsia* spp. in ticks (*6*,*7*).

Tick submissions primarily originated from eastern, central, and southern Texas, with a few from the panhandle and far western counties (Figure 2, panels A–F). The most tick submissions were from Travis County (n = 199), followed by Anderson County (n = 134). The most *A. americanum* tick submissions were from Anderson County (n = 130), and the most *D. variabilis* tick submissions were from Uvalde County (n = 35). The most *A. maculatum* (17), *I. scapularis* (n = 37), and *R. sanguineus* (n = 6) tick submissions were from Travis County. Nearly all (97.5%) *I. scapularis* submissions were from eastern Texas (east of 98°W longitude). All *A. mixtum* submissions originated from the southeastern area of the state, below 31°N latitude and east of 100°W longitude, with the most from Cameron County. The most *R. amblyommatis* ticks were from Anderson County and the most *R. tamurae* subsp. *buchneri* positive ticks were from Travis County (Figure 2, panels G–H). Ticks carrying *Candidatus* B. lonestari, *E. chaffeensis,* and *R. parkeri* were mainly from eastern and central Texas (Figure 2, panel I). Similar to our previous report, the prevalence of *Candidatus* B. lonestari (0.5%) and *E. chaffeensis* (0.4%) n *A. americanum* ticks and *R. parkeri* (5.2%) in *A. maculatum* ticks remained low and were found primarily in eastern and central Texas (*1*,*4*,*5*).

**Figure 2:**
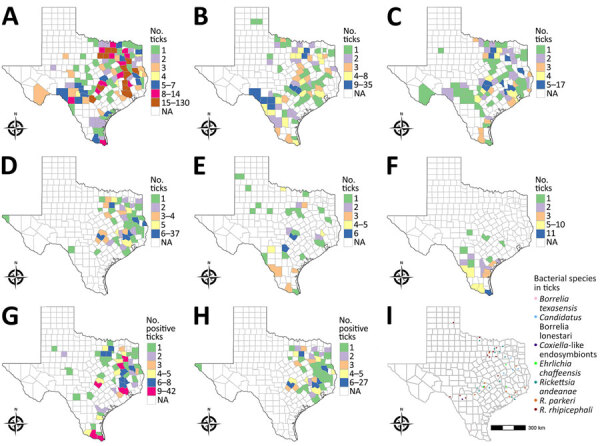
Geographic distribution of tick submissions and bacteria-positive ticks in Texas, USA, 2014–2021. A) *Amblyomma americanum* ticks. B) *Dermacentor variabilis* ticks. C) *A. maculatum* ticks. D) *Ixodes scapularis* ticks. E) *Rhipicephalus sanguineus* ticks. F) *A. mixtum* ticks. G) *R. amblyommatis* ticks. H) *R. tamurae* subspecies *buchneri* ticks. I) Bacterial species recovered from submitted ticks.

In conclusion, the temporal and geographic patterns of human–tick encounters and tickborne pathogens provide valuable guidance for outdoor activities and public health planning. The overall bacterial prevalence rate (26%) in ticks was slightly higher than our previous report (23%), highlighting the need for continued tick pathogen surveillance in this region.

Appendix Additional information about surveillance of ticks and tickborne *Borrelia*, *Ehrlichia*, and *Rickettsia* spp., Texas, USA, 2014–2021.
