# Supplementary material for: Surveillance of Ticks and Tickborne Borrelia, Ehrlichia, and Rickettsia spp., Texas, USA, 2014–2021
Source: Emerg Infect Dis. 2026 Aug;32(8):1367–70. doi: 10.3201/eid3208.251104 (PMC13426887; doi:10.3201/eid3208.251104)
Supplement: Appendix — Additional information about surveillance of ticks and tickborne Borrelia, Ehrlichia, and Rickettsia spp., Texas, USA, 2014–2021. [file 25-1104-Techapp-s1.pdf]

Article DOI: https://doi.org/10.3201/eid3208.251104

EID cannot ensure accessibility for supplementary materials supplied by authors. Readers who have difficulty accessing supplementary content should contact the authors for assistance.

Surveillance of Ticks and Tickborne *Borrelia*, *Ehrlichia*, and *Rickettsia* spp., Texas, USA, 2014–2021

Appendix

Appendix Table 1. Number of ticks positive for tested bacteria, Texas, USA, from October 1, 2014 through September 30, 2021

		No. positive								
						Rickettsia				
Tick species	Sex/stage	*"Candidatus* Borrelia lonestari*"*	*B. texasensis*	*Coxiella-like endosymbiont*	*Ehrlichia chaffeensis*	*amblyommatis*	*andeanae*	*tamurae* subsp. *buchneri*	*parkeri*	*rhipicephali*
Amblyomma americanum	Female (229)	1	0	0	1	64	0	0	0	0
	Male (190)	3	0	0	2	48	0	0	0	0
	Nymph (340)	0	0	0	0	95	0	0	0	0
	Larva (17)	0	0	0	0	1	0	0	0	0
	Undetermined* (18)	0	0	0	0	5	0	0	0	0
A. maculatum	Female (86)	0	0	0	0	0	10	0	2	0
	Male (99)	0	0	0	0	1	3	0	7	0
	Nymph (9)	0	0	0	0	0	0	0	1	0
A. mixtum	Female (7)	0	0	0	0	4	0	0	0	0
	Male (7)	0	0	0	0	3	0	0	0	0
	Nymph (33)	0	0	0	0	9	0	0	0	0
	Larva (2)	0	0	0	0	1	0	0	0	0
	Undetermined (6)	0	0	0	0	2	0	0	0	0
Dermacentor variabilis	Female (215)	0	0	0	0	1	0	0	0	10
	Male (121)	0	1	0	0	1	0	0	0	4
	Nymph (9)	0	0	0	0	0	0	0	0	0
Ixodes scapularis	Female (147)	0	0	0	0	0	0	135	0	0
	Male (7)	0	0	0	0	0	0	0	0	0
	Nymph (5)	0	0	0	0	0	0	0	0	0
	Undetermined (3)	0	0	0	0	0	0	3	0	0
Rhipicephalus sanguineus	Female (28)	0	0	1	0	1	0	0	0	0
	Male (27)	0	0	0	0	0	0	0	0	0
	Nymph (4)	0	0	0	0	0	0	0	0	0
	Undetermined (3)	0	0	0	0	0	0	0	0	0
Total	1612	4	1	1	3	236	13	138	10	14

*Undetermined specimens were damaged or otherwise not intact ticks; therefore, sex and life stage could not be determined. Not included were 1 male and 1 female *D. andersoni* positive for *R. peacockii*, and 1 *I. pacificus* positive for the rickettsial endosymbiont of that species. These samples were reported to have originated in Texas, but the native ranges for these species are outside of the state and their origins could not be independently confirmed. Other species tested but without detected bacterial agents included: 7 *Otobius megnini* (2 females, 2 nymphs, 1 undetermined), 2 *I. woodi* nymphs, and 1 *A. inornatum* nymph.

Appendix Table 2. Pairwise Tukey-type nonparametric analysis of monthly submission variations in Texas, USA, 2014 – 2021*

Tick	Comparison	Estimator (95% CI)	Statistic	p value
Adult *Amblyomma americanum*	Apr vs. Aug	0.625 (0.093 – 0.964)	0.6089303	1
	Apr vs. Dec	0.014 (0 – 0.623)	−2.9723064	0.1451475
	Apr vs. Feb	0.017 (0 – 0.668)	−2.8352001	0.2142925
	Apr vs. Jan	0.001 (0 – 0.003)	−18.385214	0
	Apr vs. Jul	0.958 (0.257 – 0.999)	2.4811721	0.4815167
	Apr vs. Jun	0.999 (0.997 – 1)	18.385214	0
	Apr vs. Mar	0.167 (0.01 – 0.801)	−1.7816777	0.9698653
	Apr vs. May	0.931 (0.203 – 0.999)	2.1757898	0.7608593
	Apr vs. Nov	0.014 (0 – 0.623)	−2.9723064	0.1481105
	Apr vs. Oct	0.001 (0 – 0.003)	−18.385214	0
	Apr vs. Sep	0.001 (0 – 0.003)	−18.385214	0
	Aug vs. Dec	0.042 (0.001 – 0.743)	−2.4811721	0.4816654
	Aug vs. Feb	0.05 (0.001 – 0.784)	−2.3097259	0.6412251
	Aug vs. Jan	0.014 (0 – 0.623)	−2.9723064	0.1457109
	Aug vs. Jul	0.694 (0.125 – 0.973)	0.986671	0.9999999
	Aug vs. Jun	0.806 (0.152 – 0.99)	1.5039947	0.9979785
	Aug vs. Mar	0.153 (0.009 – 0.786)	−1.8866244	0.9382586
	Aug vs. May	0.681 (0.122 – 0.97)	0.921076	1
	Aug vs. Nov	0.042 (0.001 – 0.743)	−2.4811721	0.48208
	Aug vs. Oct	0.014 (0 – 0.623)	−2.9723064	0.1462426
	Aug vs. Sep	0.001 (0 – 0.003)	−18.385214	0
	Dec vs. Feb	0.517 (0.159 – 0.858)	0.127942	1
	Dec vs. Jan	0.486 (0.162 – 0.823)	−0.1161878	1
	Dec vs. Jul	0.999 (0.997 – 1)	18.385214	0
	Dec vs. Jun	0.999 (0.997 – 1)	18.385214	0
	Dec vs. Mar	0.819 (0.195 – 0.988)	1.7137892	0.9824221
	Dec vs. May	0.999 (0.997 – 1)	18.385214	0
	Dec vs. Nov	0.5 (0.173 – 0.827)	0	1
	Dec vs. Oct	0.486 (0.162 – 0.823)	−0.1161878	1
	Dec vs. Sep	0.417 (0.186 – 0.691)	−0.9813774	0.9999999
	Feb vs. Jan	0.467 (0.127 – 0.84)	−0.2473338	1
	Feb vs. Jul	0.999 (0.997 – 1)	18.385214	0
	Feb vs. Jun	0.999 (0.997 – 1)	18.385214	0
	Feb vs. Mar	0.8 (0.159 – 0.988)	1.5088181	0.9978435
	Feb vs. May	0.999 (0.997 – 1)	18.385214	0
	Feb vs. Nov	0.483 (0.142 – 0.841)	−0.127942	1
	Feb vs. Oct	0.467 (0.127 – 0.84)	−0.2473338	1
	Feb vs. Sep	0.4 (0.143 – 0.727)	−0.9731163	0.9999999
	Jan vs. Jul	0.999 (0.997 – 1)	18.385214	0
	Jan vs. Jun	0.999 (0.997 – 1)	18.385214	0
	Jan vs. Mar	0.861 (0.245 – 0.992)	2.0559302	0.8504167
	Jan vs. May	0.999 (0.997 – 1)	18.385214	0
	Jan vs. Nov	0.514 (0.177 – 0.838)	0.1161878	1
	Jan vs. Oct	0.5 (0.173 – 0.827)	0	1
	Jan vs. Sep	0.417 (0.186 – 0.691)	−0.9813774	0.9999999
	Jul vs. Jun	0.653 (0.134 – 0.958)	0.838921	1
	Jul vs. Mar	0.001 (0 – 0.003)	−18.385214	0
	Jul vs. May	0.472 (0.069 – 0.915)	−0.1484348	1
	Jul vs. Nov	0.001 (0 – 0.003)	−18.385214	0
	Jul vs. Oct	0.001 (0 – 0.003)	−18.385214	0
	Jul vs. Sep	0.001 (0 – 0.003)	−18.385214	0
	Jun vs. Mar	0.001 (0 – 0.003)	−18.385214	0
	Jun vs. May	0.306 (0.032 – 0.852)	−1.0600566	0.9999994
	Jun vs. Nov	0.001 (0 – 0.003)	−18.385214	0
	Jun vs. Oct	0.001 (0 – 0.003)	−18.385214	0
	Jun vs. Sep	0.001 (0 – 0.003)	−18.385214	0
	Mar vs. May	0.986 (0.377 – 1)	2.9723064	0.14564
	Mar vs. Nov	0.181 (0.012 – 0.805)	−1.7137892	0.9824437
	Mar vs. Oct	0.139 (0.008 – 0.755)	−2.0559302	0.8500948
	Mar vs. Sep	0.083 (0.002 – 0.773)	−2.1980707	0.7406097
	May vs. Nov	0.001 (0 – 0.003)	−18.385214	0
	May vs. Oct	0.001 (0 – 0.003)	−18.385214	0
	May vs. Sep	0.001 (0 – 0.003)	−18.385214	0
	Nov vs. Oct	0.486 (0.162 – 0.823)	−0.1161878	1
	Nov vs. Sep	0.417 (0.186 – 0.691)	−0.9813774	0.9999999
	Oct vs. Sep	0.417 (0.186 – 0.691)	−0.9813774	0.9999999
Adult *A. Maculatum*	Apr vs. Aug	0.999 (0.997 – 1)	18.385214	0
	Apr vs. Dec	0.306 (0.052 – 0.78)	−1.2748391	0.9987003

Tick	Comparison	Estimator (95% CI)	Statistic	p value
	Apr vs. Feb	0.25 (0.046 – 0.697)	−1.8424288	0.8877248
	Apr vs. Jan	0.347 (0.062 – 0.81)	−0.9822576	0.9999868
	Apr vs. Jul	0.597 (0.114 – 0.945)	0.5232391	1
	Apr vs. Jun	0.694 (0.15 – 0.967)	1.0423385	0.999959
	Apr vs. Mar	0.389 (0.069 – 0.844)	−0.6835926	1
	Apr vs. May	0.556 (0.11 – 0.927)	0.3120622	1
	Apr vs. Nov	0.389 (0.069 – 0.844)	−0.6835926	1
	Apr vs. Oct	0.847 (0.18 – 0.993)	1.7214433	0.9394608
	Apr vs. Sep	0.875 (0.142 – 0.997)	1.6840624	0.9516795
	Aug vs. Dec	0.001 (0 – 0.003)	−18.385214	0
	Aug vs. Feb	0.001 (0 – 0.003)	−18.385214	0
	Aug vs. Jan	0.001 (0 – 0.003)	−18.385214	0
	Aug vs. Jul	0.001 (0 – 0.003)	−18.385214	0
	Aug vs. Jun	0.001 (0 – 0.003)	−18.385214	0
	Aug vs. Mar	0.001 (0 – 0.003)	−18.385214	0
	Aug vs. May	0.001 (0 – 0.003)	−18.385214	0
	Aug vs. Nov	0.001 (0 – 0.003)	−18.385214	0
	Aug vs. Oct	0.014 (0 – 0.596)	−2.9723064	0.1175967
	Aug vs. Sep	0.417 (0.062 – 0.886)	−0.4575696	1
	Dec vs. Feb	0.417 (0.19 – 0.685)	−0.9813774	0.9999872
	Dec vs. Jan	0.514 (0.183 – 0.833)	0.1161878	1
	Dec vs. Jul	0.861 (0.259 – 0.991)	2.0559302	0.7470246
	Dec vs. Jun	0.889 (0.235 – 0.995)	2.0665656	0.7373674
	Dec vs. Mar	0.597 (0.196 – 0.9)	0.7066701	1
	Dec vs. May	0.792 (0.241 – 0.979)	1.7422082	0.9320257
	Dec vs. Nov	0.597 (0.196 – 0.9)	0.7066701	1
	Dec vs. Oct	0.999 (0.997 – 1)	18.385214	0
	Dec vs. Sep	0.903 (0.198 – 0.997)	1.9915998	0.7952506
	Feb vs. Jan	0.583 (0.315 – 0.81)	0.9813774	0.9999871
	Feb vs. Jul	0.917 (0.243 – 0.997)	2.1980707	0.628237
	Feb vs. Jun	0.917 (0.243 – 0.997)	2.1980707	0.6283202
	Feb vs. Mar	0.667 (0.301 – 0.903)	1.4612826	0.9907733
	Feb vs. May	0.833 (0.299 – 0.983)	2.1206206	0.6935876
	Feb vs. Nov	0.667 (0.301 – 0.903)	1.4612826	0.9907523
	Feb vs. Oct	0.999 (0.997 – 1)	18.385214	0
	Feb vs. Sep	0.917 (0.243 – 0.997)	2.1980707	0.6279683
	Jan vs. Jul	0.764 (0.137 – 0.985)	1.2618714	0.9988849
	Jan vs. Jun	0.792 (0.171 – 0.986)	1.4847824	0.9886049
	Jan vs. Mar	0.556 (0.163 – 0.889)	0.388996	1
	Jan vs. May	0.694 (0.156 – 0.966)	1.0600566	0.9999438
	Jan vs. Nov	0.556 (0.163 – 0.889)	0.388996	1
	Jan vs. Oct	0.917 (0.203 – 0.998)	2.0647279	0.740863
	Jan vs. Sep	0.903 (0.198 – 0.997)	1.9915998	0.7951977
	Jul vs. Jun	0.681 (0.149 – 0.963)	0.9797698	0.9999875
	Jul vs. Mar	0.25 (0.025 – 0.81)	−1.3964445	0.9950194
	Jul vs. May	0.472 (0.079 – 0.903)	−0.1536203	1
	Jul vs. Nov	0.25 (0.025 – 0.81)	−1.3964445	0.994971
	Jul vs. Oct	0.917 (0.29 – 0.997)	2.3611066	0.4854958
	Jul vs. Sep	0.847 (0.106 – 0.996)	1.4453134	0.9921194
	Jun vs. Mar	0.167 (0.012 – 0.774)	−1.8365114	0.8910904
	Jun vs. May	0.278 (0.028 – 0.836)	−1.1976715	0.999526
	Jun vs. Nov	0.167 (0.012 – 0.774)	−1.8365114	0.8902984
	Jun vs. Oct	0.736 (0.174 – 0.974)	1.2863877	0.9984838
	Jun vs. Sep	0.847 (0.106 – 0.996)	1.4453134	0.9920615
	Mar vs. May	0.694 (0.181 – 0.959)	1.1411943	0.9997909
	Mar vs. Nov	0.5 (0.122 – 0.878)	0	1
	Mar vs. Oct	0.972 (0.404 – 0.999)	2.9213504	0.1345192
	Mar vs. Sep	0.889 (0.166 – 0.997)	1.825712	0.8959676
	May vs. Nov	0.306 (0.041 – 0.819)	−1.1411943	0.9997923
	May vs. Oct	0.917 (0.415 – 0.994)	2.837697	0.1681093
	May vs. Sep	0.861 (0.122 – 0.996)	1.5587194	0.9793934
	Nov vs. Oct	0.972 (0.404 – 0.999)	2.9213504	0.1339627
	Nov vs. Sep	0.889 (0.166 – 0.997)	1.825712	0.8957741
	Oct vs. Sep	0.833 (0.093 – 0.996)	1.3411983	0.9972083
Adult *Dermacentor variabilis*	Apr vs. Aug	0.722 (0.114 – 0.981)	1.0403522	0.9999821
	Apr vs. Dec	0.042 (0.001 – 0.647)	−2.743039	0.2372742
	Apr vs. Feb	0.083 (0.002 – 0.819)	−2.0065548	0.8135218
	Apr vs. Jan	0.097 (0.004 – 0.761)	−2.1526092	0.7029153
	Apr vs. Jul	0.417 (0.065 – 0.881)	−0.4714384	1

Tick	Comparison	Estimator (95% CI)	Statistic	p value
	Apr vs. Jun	0.999 (0.997 – 1)	18.385214	0
	Apr vs. Mar	0.333 (0.036 – 0.87)	−0.8745858	0.9999996
	Apr vs. May	0.847 (0.136 – 0.995)	1.5745099	0.9831955
	Apr vs. Nov	0.194 (0.012 – 0.833)	−1.536813	0.9875668
	Apr vs. Oct	0.722 (0.132 – 0.978)	1.1022084	0.9999414
	Apr vs. Sep	0.333 (0.036 – 0.87)	−0.8745858	0.9999996
	Aug vs. Dec	0.001 (0 – 0.003)	−18.385214	0
	Aug vs. Feb	0.067 (0.003 – 0.61)	−2.7979165	0.203316
	Aug vs. Jan	0.056 (0.001 – 0.78)	−2.2614744	0.6076983
	Aug vs. Jul	0.25 (0.017 – 0.862)	−1.2257663	0.9995958
	Aug vs. Jun	0.999 (0.997 – 1)	18.385214	0
	Aug vs. Mar	0.306 (0.029 – 0.868)	−0.9940804	0.9999928
	Aug vs. May	0.597 (0.127 – 0.938)	0.5555511	1
	Aug vs. Nov	0.139 (0.01 – 0.723)	−2.1460132	0.7086489
	Aug vs. Oct	0.556 (0.101 – 0.933)	0.3031866	1
	Aug vs. Sep	0.306 (0.029 – 0.868)	−0.9940804	0.9999928
	Dec vs. Feb	0.65 (0.119 – 0.962)	0.7723315	1
	Dec vs. Jan	0.458 (0.088 – 0.881)	−0.2520111	1
	Dec vs. Jul	0.958 (0.353 – 0.999)	2.743039	0.238864
	Dec vs. Jun	0.999 (0.997 – 1)	18.385214	0
	Dec vs. Mar	0.833 (0.193 – 0.991)	1.7314795	0.9494822
	Dec vs. May	0.999 (0.997 – 1)	18.385214	0
	Dec vs. Nov	0.917 (0.409 – 0.994)	2.837697	0.186938
	Dec vs. Oct	0.999 (0.997 – 1)	18.385214	0
	Dec vs. Sep	0.833 (0.193 – 0.991)	1.7314795	0.9504287
	Feb vs. Jan	0.383 (0.051 – 0.878)	−0.6360276	1
	Feb vs. Jul	0.883 (0.236 – 0.995)	2.0718993	0.768024
	Feb vs. Jun	0.999 (0.997 – 1)	18.385214	0
	Feb vs. Mar	0.75 (0.167 – 0.978)	1.3281222	0.9984978
	Feb vs. May	0.967 (0.354 – 0.999)	2.7759182	0.2188814
	Feb vs. Nov	0.733 (0.17 – 0.974)	1.2747384	0.9992248
	Feb vs. Oct	0.967 (0.354 – 0.999)	2.7759182	0.219014
	Feb vs. Sep	0.75 (0.167 – 0.978)	1.3281222	0.9984764
	Jan vs. Jul	0.889 (0.216 – 0.996)	2.0197694	0.8025488
	Jan vs. Jun	0.999 (0.997 – 1)	18.385214	0
	Jan vs. Mar	0.778 (0.184 – 0.982)	1.4946172	0.9914548
	Jan vs. May	0.972 (0.231 – 1)	2.4441771	0.4491972
	Jan vs. Nov	0.819 (0.136 – 0.992)	1.4733908	0.9929574
	Jan vs. Oct	0.944 (0.22 – 0.999)	2.2614744	0.6107055
	Jan vs. Sep	0.778 (0.184 – 0.982)	1.4946172	0.9914643
	Jul vs. Jun	0.999 (0.997 – 1)	18.385214	0
	Jul vs. Mar	0.375 (0.048 – 0.878)	−0.6723038	1
	Jul vs. May	0.875 (0.131 – 0.997)	1.6601738	0.9685141
	Jul vs. Nov	0.25 (0.023 – 0.822)	−1.3676487	0.9976518
	Jul vs. Oct	0.764 (0.146 – 0.984)	1.3055225	0.9988424
	Jul vs. Sep	0.375 (0.048 – 0.878)	−0.6723038	1
	Jun vs. Mar	0.028 (0.001 – 0.605)	−2.9213504	0.150248
	Jun vs. May	0.069 (0.002 – 0.787)	−2.1757898	0.6841285
	Jun vs. Nov	0.001 (0 – 0.003)	−18.385214	0
	Jun vs. Oct	0.028 (0.001 – 0.605)	−2.9213504	0.1511605
	Jun vs. Sep	0.028 (0.001 – 0.605)	−2.9213504	0.1505158
	Mar vs. May	0.792 (0.159 – 0.987)	1.4544775	0.9940658
	Mar vs. Nov	0.417 (0.056 – 0.896)	−0.4418408	1
	Mar vs. Oct	0.736 (0.154 – 0.977)	1.2284992	0.9995811
	Mar vs. Sep	0.5 (0.087 – 0.913)	0	1
	May vs. Nov	0.069 (0.002 – 0.76)	−2.2658603	0.6065876
	May vs. Oct	0.431 (0.064 – 0.893)	−0.3812323	1
	May vs. Sep	0.208 (0.013 – 0.841)	−1.4544775	0.9940876
	Nov vs. Oct	0.903 (0.276 – 0.996)	2.2845217	0.5905807
	Nov vs. Sep	0.583 (0.104 – 0.944)	0.4418408	1
	Oct vs. Sep	0.264 (0.023 – 0.846)	−1.2284992	0.9995764
Adult *Ixodes scapularis*	Apr vs. Aug	0.056 (0.003 – 0.564)	−2.9916494	0.1139027
	Apr vs. Dec	0.444 (0.06 – 0.91)	−0.2874937	1
	Apr vs. Feb	0.383 (0.045 – 0.89)	−0.6040879	1
	Apr vs. Jan	0.556 (0.108 – 0.928)	0.3120622	1
	Apr vs. Jul	0.028 (0.001 – 0.603)	−2.9213504	0.1377616
	Apr vs. Jun	0.083 (0.006 – 0.589)	−2.837697	0.1712152
	Apr vs. Mar	0.694 (0.143 – 0.969)	1.0254802	0.9998219
	Apr vs. May	0.375 (0.049 – 0.876)	−0.6772655	0.9999998

Tick	Comparison	Estimator (95% CI)	Statistic	p value
	Apr vs. Nov	0.194 (0.017 – 0.77)	−1.7659234	0.8998664
	Apr vs. Oct	0.083 (0.006 – 0.589)	−2.837697	0.1742254
	Apr vs. Sep	0.028 (0.001 – 0.603)	−2.9213504	0.1364609
	Aug vs. Dec	0.972 (0.397 – 0.999)	2.9213504	0.1375914
	Aug vs. Feb	0.867 (0.125 – 0.997)	1.6023232	0.9571399
	Aug vs. Jan	0.944 (0.436 – 0.997)	2.9916494	0.1143178
	Aug vs. Jul	0.417 (0.105 – 0.813)	−0.6086244	1
	Aug vs. Jun	0.583 (0.151 – 0.917)	0.5322266	1
	Aug vs. Mar	0.999 (0.997 – 1)	18.385214	0
	Aug vs. May	0.861 (0.214 – 0.993)	1.9052867	0.8268876
	Aug vs. Nov	0.722 (0.197 – 0.965)	1.3231902	0.9944956
	Aug vs. Oct	0.583 (0.151 – 0.917)	0.5322266	1
	Aug vs. Sep	0.417 (0.105 – 0.813)	−0.6086244	1
	Dec vs. Feb	0.483 (0.074 – 0.916)	−0.0884959	1
	Dec vs. Jan	0.597 (0.101 – 0.951)	0.4991559	1
	Dec vs. Jul	0.014 (0 – 0.604)	−2.9723064	0.1181909
	Dec vs. Jun	0.042 (0.001 – 0.645)	−2.743039	0.2174302
	Dec vs. Mar	0.694 (0.159 – 0.965)	1.07871	0.9996287
	Dec vs. May	0.417 (0.065 – 0.88)	−0.4714384	1
	Dec vs. Nov	0.222 (0.019 – 0.806)	−1.5287193	0.9726925
	Dec vs. Oct	0.042 (0.001 – 0.645)	−2.743039	0.2178826
	Dec vs. Sep	0.014 (0 – 0.604)	−2.9723064	0.1190233
	Feb vs. Jan	0.65 (0.119 – 0.962)	0.7726543	0.9999982
	Feb vs. Jul	0.117 (0.003 – 0.847)	−1.7702315	0.8993243
	Feb vs. Jun	0.15 (0.004 – 0.896)	−1.4582966	0.9830781
	Feb vs. Mar	0.75 (0.178 – 0.977)	1.3632047	0.9921437
	Feb vs. May	0.467 (0.069 – 0.912)	−0.1763213	1
	Feb vs. Nov	0.283 (0.024 – 0.866)	−1.0844091	0.9995857
	Feb vs. Oct	0.15 (0.004 – 0.896)	−1.4582966	0.9830958
	Feb vs. Sep	0.117 (0.003 – 0.847)	−1.7702315	0.8986163
	Jan vs. Jul	0.028 (0.001 – 0.603)	−2.9213504	0.1391251
	Jan vs. Jun	0.083 (0.006 – 0.589)	−2.837697	0.1708199
	Jan vs. Mar	0.597 (0.106 – 0.949)	0.509063	1
	Jan vs. May	0.347 (0.041 – 0.868)	−0.8195835	0.9999949
	Jan vs. Nov	0.181 (0.014 – 0.77)	−1.8151831	0.8777567
	Jan vs. Oct	0.083 (0.006 – 0.589)	−2.837697	0.1716117
	Jan vs. Sep	0.028 (0.001 – 0.603)	−2.9213504	0.1394084
	Jul vs. Jun	0.667 (0.205 – 0.939)	1.1046258	0.9994742
	Jul vs. Mar	0.999 (0.997 – 1)	18.385214	0
	Jul vs. May	0.889 (0.23 – 0.995)	2.0665656	0.71597
	Jul vs. Nov	0.778 (0.238 – 0.975)	1.6929592	0.9292413
	Jul vs. Oct	0.667 (0.205 – 0.939)	1.1046258	0.9994742
	Jul vs. Sep	0.5 (0.177 – 0.823)	0	1
	Jun vs. Mar	0.999 (0.997 – 1)	18.385214	0
	Jun vs. May	0.833 (0.194 – 0.99)	1.7314795	0.9152694
	Jun vs. Nov	0.667 (0.164 – 0.953)	0.9741884	0.9999158
	Jun vs. Oct	0.5 (0.113 – 0.887)	0	1
	Jun vs. Sep	0.333 (0.061 – 0.795)	−1.1046258	0.9994855
	Mar vs. May	0.208 (0.019 – 0.781)	−1.6710674	0.9368932
	Mar vs. Nov	0.097 (0.004 – 0.722)	−2.2845217	0.5423626
	Mar vs. Oct	0.001 (0 – 0.003)	−18.385214	0
	Mar vs. Sep	0.001 (0 – 0.003)	−18.385214	0
	May vs. Nov	0.319 (0.039 – 0.846)	−1.0048605	0.9998669
	May vs. Oct	0.167 (0.01 – 0.806)	−1.7314795	0.9151938
	May vs. Sep	0.111 (0.005 – 0.77)	−2.0665656	0.714033
	Nov vs. Oct	0.333 (0.047 – 0.836)	−0.9741884	0.9999188
	Nov vs. Sep	0.222 (0.025 – 0.762)	−1.6929592	0.9302843
	Oct vs. Sep	0.333 (0.061 – 0.795)	−1.1046258	0.9994787
Rickettsia amblyommatis positive ticks	Apr vs. Aug	0.667 (0.12 – 0.967)	0.8436719	0.9999999
	Apr vs. Dec	0.042 (0.001 – 0.647)	−2.743039	0.2323628
	Apr vs. Feb	0.001 (0 – 0.003)	−18.255282	0
	Apr vs. Jan	0.001 (0 – 0.003)	−18.255282	0
	Apr vs. Jul	0.833 (0.193 – 0.991)	1.7314795	0.9529715
	Apr vs. Jun	0.958 (0.353 – 0.999)	2.743039	0.2326235
	Apr vs. Mar	0.125 (0.003 – 0.862)	−1.6840624	0.9654063
	Apr vs. May	0.999 (0.997 – 1)	18.255282	0
	Apr vs. Nov	0.25 (0.011 – 0.909)	−1.0567054	0.9999842
	Apr vs. Oct	0.333 (0.033 – 0.88)	−0.8436719	0.9999999
	Apr vs. Sep	0.333 (0.033 – 0.88)	−0.8436719	0.9999999

Tick	Comparison	Estimator (95% CI)	Statistic	p value
	Aug vs. Dec	0.042 (0.001 – 0.731)	−2.4811721	0.416005
	Aug vs. Feb	0.001 (0 – 0.003)	−18.255282	0
	Aug vs. Jan	0.001 (0 – 0.003)	−18.255282	0
	Aug vs. Jul	0.583 (0.101 – 0.946)	0.4359883	1
	Aug vs. Jun	0.653 (0.107 – 0.967)	0.7489644	1
	Aug vs. Mar	0.083 (0.003 – 0.741)	−2.2752193	0.5952835
	Aug vs. May	0.681 (0.104 – 0.975)	0.8499622	0.9999999
	Aug vs. Nov	0.167 (0.009 – 0.82)	−1.6830853	0.9657478
	Aug vs. Oct	0.208 (0.018 – 0.794)	−1.6282124	0.9767596
	Aug vs. Sep	0.236 (0.023 – 0.804)	−1.4865642	0.9930802
	Dec vs. Feb	0.417 (0.189 – 0.687)	−0.9813774	0.9999968
	Dec vs. Jan	0.417 (0.189 – 0.687)	−0.9813774	0.9999969
	Dec vs. Jul	0.986 (0.394 – 1)	2.9723064	0.124718
	Dec vs. Jun	0.999 (0.997 – 1)	18.255282	0
	Dec vs. Mar	0.583 (0.183 – 0.897)	0.6009684	1
	Dec vs. May	0.999 (0.997 – 1)	18.255282	0
	Dec vs. Nov	0.633 (0.175 – 0.934)	0.8514025	0.9999999
	Dec vs. Oct	0.694 (0.217 – 0.949)	1.2748391	0.9994045
	Dec vs. Sep	0.833 (0.221 – 0.989)	1.8365114	0.9129487
	Feb vs. Jan	0.5 (0.499 – 0.501)	0	1
	Feb vs. Jul	0.999 (0.997 – 1)	18.255282	0
	Feb vs. Jun	0.999 (0.997 – 1)	18.255282	0
	Feb vs. Mar	0.667 (0.298 – 0.904)	1.4612826	0.994583
	Feb vs. May	0.999 (0.997 – 1)	18.255282	0
	Feb vs. Nov	0.7 (0.257 – 0.94)	1.4528132	0.9950453
	Feb vs. Oct	0.75 (0.299 – 0.955)	1.8424288	0.9111255
	Feb vs. Sep	0.917 (0.237 – 0.997)	2.1980707	0.6629311
	Jan vs. Jul	0.999 (0.997 – 1)	18.255282	0
	Jan vs. Jun	0.999 (0.997 – 1)	18.255282	0
	Jan vs. Mar	0.667 (0.298 – 0.904)	1.4612826	0.994635
	Jan vs. May	0.999 (0.997 – 1)	18.255282	0
	Jan vs. Nov	0.7 (0.257 – 0.94)	1.4528132	0.9950632
	Jan vs. Oct	0.75 (0.299 – 0.955)	1.8424288	0.9101504
	Jan vs. Sep	0.917 (0.237 – 0.997)	2.1980707	0.6628611
	Jul vs. Jun	0.611 (0.121 – 0.947)	0.6083894	1
	Jul vs. Mar	0.042 (0.001 – 0.731)	−2.4811721	0.4163595
	Jul vs. May	0.583 (0.104 – 0.944)	0.4418408	1
	Jul vs. Nov	0.083 (0.002 – 0.771)	−2.1721593	0.6860397
	Jul vs. Oct	0.111 (0.005 – 0.742)	−2.1708269	0.6852543
	Jul vs. Sep	0.111 (0.005 – 0.742)	−2.1708269	0.6853395
	Jun vs. Mar	0.014 (0 – 0.606)	−2.9723064	0.1271077
	Jun vs. May	0.486 (0.079 – 0.913)	−0.0755972	1
	Jun vs. Nov	0.033 (0 – 0.805)	−2.3016698	0.5722345
	Jun vs. Oct	0.042 (0.001 – 0.731)	−2.4811721	0.4151937
	Jun vs. Sep	0.042 (0.001 – 0.731)	−2.4811721	0.4142469
	Mar vs. May	0.999 (0.997 – 1)	18.255282	0
	Mar vs. Nov	0.567 (0.12 – 0.926)	0.3875669	1
	Mar vs. Oct	0.625 (0.156 – 0.937)	0.760736	1
	Mar vs. Sep	0.764 (0.187 – 0.978)	1.4537683	0.9950072
	May vs. Nov	0.017 (0 – 0.652)	−2.8352001	0.1830927
	May vs. Oct	0.014 (0 – 0.606)	−2.9723064	0.1252244
	May vs. Sep	0.014 (0 – 0.606)	−2.9723064	0.1259856
	Nov vs. Oct	0.55 (0.107 – 0.926)	0.282337	1
	Nov vs. Sep	0.65 (0.106 – 0.967)	0.7360259	1
	Oct vs. Sep	0.583 (0.109 – 0.941)	0.4510789	1
R. tamurae subsp. *buchneri* positive ticks	Apr vs. Aug	0.014 (0 – 0.607)	−2.9723064	0.1245006
	Apr vs. Dec	0.375 (0.044 – 0.886)	−0.6534943	1
	Apr vs. Feb	0.25 (0.021 – 0.836)	−1.3197667	0.9966454
	Apr vs. Jan	0.444 (0.068 – 0.898)	−0.3053343	1
	Apr vs. Jul	0.001 (0 – 0.003)	−18.255282	0
	Apr vs. Jun	0.042 (0.001 – 0.647)	−2.743039	0.2219066
	Apr vs. Mar	0.556 (0.101 – 0.933)	0.3031866	1
	Apr vs. May	0.319 (0.035 – 0.857)	−0.9718149	0.9999723
	Apr vs. Nov	0.133 (0.007 – 0.78)	−1.9520498	0.8149908
	Apr vs. Oct	0.028 (0.001 – 0.606)	−2.9213504	0.1399429
	Apr vs. Sep	0.014 (0 – 0.607)	−2.9723064	0.1211915
	Aug vs. Dec	0.986 (0.393 – 1)	2.9723064	0.1187612
	Aug vs. Feb	0.867 (0.185 – 0.995)	1.8262336	0.8877455
	Aug vs. Jan	0.972 (0.394 – 0.999)	2.9213504	0.1392422

Tick	Comparison	Estimator (95% CI)	Statistic	p value
	Aug vs. Jul	0.417 (0.189 – 0.687)	−0.9813774	0.9999674
	Aug vs. Jun	0.667 (0.204 – 0.94)	1.1046258	0.999765
	Aug vs. Mar	0.999 (0.997 – 1)	18.255282	0
	Aug vs. May	0.889 (0.229 – 0.995)	2.0665656	0.7330436
	Aug vs. Nov	0.85 (0.203 – 0.992)	1.8313923	0.8857055
	Aug vs. Oct	0.583 (0.186 – 0.895)	0.6086244	1
	Aug vs. Sep	0.5 (0.176 – 0.824)	0	1
	Dec vs. Feb	0.35 (0.041 – 0.871)	−0.8002273	0.9999992
	Dec vs. Jan	0.583 (0.101 – 0.946)	0.4359883	1
	Dec vs. Jul	0.001 (0 – 0.003)	−18.255282	0
	Dec vs. Jun	0.042 (0.001 – 0.647)	−2.743039	0.2229854
	Dec vs. Mar	0.625 (0.136 – 0.946)	0.7095341	0.9999999
	Dec vs. May	0.417 (0.065 – 0.881)	−0.4714384	1
	Dec vs. Nov	0.2 (0.018 – 0.77)	−1.7505026	0.9215992
	Dec vs. Oct	0.028 (0.001 – 0.606)	−2.9213504	0.1418804
	Dec vs. Sep	0.014 (0 – 0.607)	−2.9723064	0.120387
	Feb vs. Jan	0.683 (0.13 – 0.969)	0.9427386	0.9999838
	Feb vs. Jul	0.1 (0.003 – 0.809)	−1.9775021	0.7983548
	Feb vs. Jun	0.2 (0.011 – 0.85)	−1.4546683	0.9886682
	Feb vs. Mar	0.783 (0.208 – 0.98)	1.6049047	0.9659294
	Feb vs. May	0.567 (0.1 – 0.939)	0.3571045	1
	Feb vs. Nov		−0.609578	1
	Feb vs. Oct	0.167 (0.008 – 0.831)	−1.6459208	0.9559988
	Feb vs. Sep	0.133 (0.005 – 0.815)	−1.8262336	0.8879641
	Jan vs. Jul	0.001 (0 – 0.003)	−18.255282	0
	Jan vs. Jun	0.083 (0.006 – 0.591)	−2.837697	0.1753037
	Jan vs. Mar	0.569 (0.096 – 0.943)	0.3634513	1
	Jan vs. May	0.361 (0.046 – 0.87)	−0.7568598	0.9999997
	Jan vs. Nov	0.2 (0.014 – 0.818)	−1.572788	0.972534
	Jan vs. Oct	0.056 (0.003 – 0.567)	−2.9916494	0.1138534
	Jan vs. Sep	0.028 (0.001 – 0.606)	−2.9213504	0.1404821
	Jul vs. Jun	0.75 (0.299 – 0.955)	1.8424288	0.880802
	Jul vs. Mar	0.999 (0.997 – 1)	18.255282	0
	Jul vs. May	0.917 (0.236 – 0.997)	2.1980707	0.6272612
	Jul vs. Nov	0.9 (0.191 – 0.997)	1.9775021	0.7979514
	Jul vs. Oct	0.667 (0.297 – 0.904)	1.4612826	0.9877709
	Jul vs. Sep	0.583 (0.313 – 0.811)	0.9813774	0.9999669
	Jun vs. Mar	0.999 (0.997 – 1)	18.255282	0
	Jun vs. May	0.833 (0.192 – 0.991)	1.7314795	0.9287788
	Jun vs. Nov	0.75 (0.173 – 0.977)	1.3509344	0.9955347
	Jun vs. Oct	0.417 (0.083 – 0.85)	−0.5322266	1
	Jun vs. Sep	0.333 (0.06 – 0.796)	−1.1046258	0.9997648
	Mar vs. May	0.292 (0.035 – 0.823)	−1.1988855	0.9991548
	Mar vs. Nov	0.1 (0.007 – 0.637)	−2.6078526	0.3039023
	Mar vs. Oct	0.001 (0 – 0.003)	-18.255282	0
	Mar vs. Sep	0.001 (0 – 0.003)	-18.255282	0
	May vs. Nov	0.317 (0.035 – 0.854)	-0.9921629	0.9999598
	May vs. Oct	0.139 (0.007 – 0.788)	-1.9052867	0.8437896
	May vs. Sep	0.111 (0.005 – 0.771)	-2.0665656	0.7341227
	Nov vs. Oct	0.2 (0.015 – 0.808)	-1.6091578	0.9651719
	Nov vs. Sep	0.15 (0.008 – 0.797)	-1.8313923	0.8854224
	Oct vs. Sep	0.417 (0.105 – 0.814)	-0.6086244	1

*Data from 2020 were excluded from the temporal analysis due to COVID-19 pandemic-related testing delays.
